# Compulsory vaccination against COVID-19: a legal and ethical perspective on public good versus personal reticence

**DOI:** 10.1007/s11845-022-02942-x

**Published:** 2022-02-25

**Authors:** Colum P. Dunne, Eimear Spain

**Affiliations:** 1 School of Medicine, University of Limerick, Limerick, Ireland; 2 School of Law, University of Limerick, Limerick, Ireland

**Keywords:** Autonomy, Casuistry, Compulsory, COVID-19, Infectious disease, Mandatory, Rhetorical reasoning, Vaccination

## Abstract

Coercive measures to protect public health are controversial, eliciting questions regarding state-patient relationships and conflicts between individual autonomy and public good. This is challenging in a time when respect for patient autonomy has become elevated yet society faces an increasing number of public health challenges, the most recent being the SARS-CoV-2 virus (COVID-19). In that context, there is emphasis on increasing vaccination rates internationally in order to achieve “herd immunity”, raising the possibility of compulsory vaccination of populations in the future. Here, we explore current rights of individuals to decline vaccination, utilising prior learning from other viral pathogens internationally (specifically, measles, mumps and rubella), and related public health outcomes. Further, we consider freedom of choice versus mandatory treatment necessitated to avoid contagion during disease outbreaks (such as COVID-19). In doing so, we utilise rhetorical reasoning in the form of casuistry focusing on the core challenges regarding public good versus personal antipathy towards vaccination.

## Introduction

Coercive measures to protect public health, whether health promotion initiatives or schemes to prevent or control disease, are controversial. They elicit questions regarding state-patient relationships and conflicts between individual autonomy and the public good. This is particularly challenging in a time when respect for patient autonomy has become elevated yet society faces an increasing number of public health challenges, the most recent being the SARS-CoV-2 virus (COVID-19).

In that context, should the state seek to impose restrictions on personal liberty in the interests of the public good, it is argued that the state could justify every measure, reducing personal freedom of choice and civil liberties, in favour of public health protection. It is suggested that all interventions should be proportionate and “necessary” [1] and utilised only when minimally restrictive approaches have been exhausted.There should be objectivity in any risk assessment employed, exploring factors including the likelihood of the spread of infection, the extent of the threat posed, and the effect of inequalities and social exclusion [1]. There should also be full respect for individuals not implicated in the immediate health threat [1]. Is it arguable then that, while the law has an important role in the protection of individuals from unjustifiable interference with individual rights, “the health interest of the community can sometimes be so strong- or the threat to health so great- that even compulsory action against the bodily integrity and freedoms of individuals can be defended?” [1].

Different approaches to deal with the spread of infectious disease are taken in different jurisdictions, with states employing a range of legislative measures enabling mandatory interventions including compulsory vaccination, compulsory medical examination, compulsory quarantine, compulsory isolation, or detention of infected persons [2]. Acknowledging that there are those who advocate for compulsory medical treatment or vaccination against infectious disease, most modern western democracies have taken the view generally that to require compulsory medical treatment for an infectious disease or to require compulsory vaccination of the population against an infectious disease is a step too far; an unwarranted infringement of the right to bodily integrity, even in the face of overwhelming evidence of vaccine benefits [3]. However, the COVID-19 crisis has caused some to rethink this position, and the issue of compulsory vaccination has become a hotly contested topic. Notably, a prescient Cave argued in 2017 that globalisation “necessitates preparedness strategies for pandemics”, including compulsory vaccination as a “policy option in the context of a severe, vaccine‐preventable pandemic outbreak” [4]. By January 2022, over 330 million cases of COVID-19 have been confirmed to the World Health Organization (WHO) with over 5,500,000 deaths reported [5]. The real figure is likely to be much higher. COVID-19 has also caused major disruptions in the global economy, with the World Bank estimating that the resulting “global recession… was surpassed only by the two World Wars and the Great Depression over the past century and a half” [6], while the WHO estimates that almost 50% of the 3.3 billion global workforce are at risk of losing their livelihoods [7]. Given the impact of COVID-19 at a societal and individual level, and the potential for even more dangerous mutations emerging, it seems reasonable to suggest that traditional respect for autonomy exhibited in most western democracies could warrant review in the context of potential advantages associated with the use of compulsory vaccination employed against pandemic threats.

## Refusal of vaccinations

Even before the current pandemic, vaccine hesitancy was a recognised phenomenon, with the WHO identifying it as one of the top ten global health threats in 2019 [8]. Those who refuse to accept vaccination, most often for their children, differ according to the intensity of their distrust of immunization and their stated reasons for adopting that view. That phenomenon translates, in effect, into some parents allowing their children receive all vaccines recommended to them, while others refuse or delay engaging with some or any immunizations [9, 10]. The most prevalent reasons for refusal of vaccinations relate to concerns regarding vaccine safety and distrust of the pharmaceutical sector [11]. Vaccine uptake has also been linked to trust in government [12]. Those worries have, in part, been fueled by erroneous, incompetent, or unethical reporting of fabricated scientific results associated with a 1997 study published by Andrew Wakefield, a British surgeon [13]. The article was published in *The Lancet*, a prestigious medical journal, stating that the measles, mumps, rubella (MMR) vaccine was increasing autism in British children. That paper has since been discredited due to serious procedural errors, undisclosed financial conflict of interest, and ethical violations. Andrew Wakefield lost his medical licence, and the paper was retracted from the journal [14]. Nonetheless, the hypothesis was taken seriously, and several other major studies were conducted. None of them found a link between any vaccine and the likelihood of developing autism. The damage, however, was done [15].

The dangers of misinformation have become even more acute in the age of social media, whereby fabrications and mistruths can be spread with impunity and reach a large audience [16]. In a report published by the Centre for Countering Digital Hate (CCDH), an international not-for-profit NGO that seeks to disrupt the architecture of online hate and misinformation based in London and Washington DC, it was noted that 17 million people subscribe to anti-vaccine groups on Youtube and 31 million on Facebook, potentially amounting to US$1 billion in advertising revenues for social media firms, including $989 million to Facebook and Instagram alone [17, 18]. While social media companies have indicated a willingness to tackle misinformation from anti-vaccination groups, the CCDC notes that fewer than 1 in 20 posts containing misinformation about COVID-19 were tackled [18, 19]. In a recent survey, it was found that 1/3 of the British population were either unlikely to agree to be vaccinated against COVID-19 or were as yet undecided, with those who rely on social media for news more likely to fall into this category [19]. Meanwhile, 46% of the US population were vaccine hesitant [19] with 63% now fully vaccinated [20]. In another recent study (published in 2021) involving 13,426 people in 19 countries, 71.5% of participants reported that they would be very or somewhat likely to take a COVID-19 vaccine, ranging from almost 90% (in China) to less than 55% (in Russia) [21]. This is reflected in diverging levels of vaccine uptake across the globe ranging from 92.4% in the United Arab Emirates to 47.3% in Russia [20].

## Compulsory vaccination pre-COVID-19

Some jurisdictions made vaccination against infectious disease compulsory in different contexts, while allowing a variety of exemptions. For example, in Singapore, vaccination for diphtheria and measles is mandated by law [22]. In England and Wales, the Vaccination Act 1853 authorised compulsory vaccination of infants against smallpox. In Ireland, the Health Act of 1947 (S31 (7)) authorises the state to issue regulations requiring people to submit to measures including immunisation against a particular infectious disease; however, no such measure against the general population has yet been issued. In other jurisdictions, legislation requires children to receive vaccines, or document immunity, in order to enrol in public school [23] based on development of “herd immunity” or the resistance to the spread of a contagious disease within a population that results if a sufficiently high proportion of individuals are immune to the disease, especially through vaccination [24].

Even in those jurisdictions requiring vaccination, exemptions from vaccination are permitted to varying degrees including on the basis of personal or religious belief. The net effect is that when clusters of exemptions are accommodated, they are co-located with outbreaks of those preventable diseases [25]. This was recently exemplified when, in 2019, incidence of measles outbreaks in the USA (especially New York) escalated and was attributed specifically and unequivocally to failure of parents to vaccinate their children [26, 27]. This despite measles in the USA being officially declared as eradicated in 2000 [28]. Following a similar outbreak in California, legislation was passed eliminating personal and religious belief exemptions for vaccines [29] and limiting medical exemptions [30].

As a result of fears regarding loss of herd immunity, in 2014, certain states in the USA (e.g. Michigan) enacted legislation requiring local health departments to provide parents with education about “the risks of not receiving the vaccines being waived and the benefits of vaccination to the individual and the community” [31, 32]. Indeed, some clinicians are expanding on this concept and declaring that, in circumstances where a family member is ill or immuno-compromised, all members of that family and especially children should undergo mandatory vaccination [33]. Perhaps of greater consequence to the health of unvaccinated children, and one not anticipated by their parents, is an emerging trend of physicians refusing to care for families that decline vaccination. One US study reported as many as 40% of physicians denying care to these families, despite the US Centers for Disease Control and the American Academy of Pediatrics urging physicians not to refuse treatment for fear that children will not receive necessary care [34]. The trend, however, is persistent and international [35].

## Compulsory vaccination and casuistry

In this context, societies around the world must decide if freedom of conscience should prevail, or whether it is acceptable to demand citizens accept a vaccine against a virus that has killed over five million people, resulted in the closure of economies all over the world, and has necessitated unprecedented restrictions on public freedoms. In this article, we explore Aristotle’s concept of casuistry (*casus* is Latin for “case” or “occurrence”) that became synonymous with the Catholic Jesuit Order’s theology and both promotion of personal responsibility and respect of freedom of conscience, as an alternative approach to ascertain whether we as a society should require compulsory vaccination of the population against COVID-19. Rather than appealing to principles to solve a problem, that is, a reliance on principles derived from old problems to solve a new problem, casuistry requires an exploration of analogous scenarios in an effort to find commonalities and discordance, in order to elucidate the problem and determine a solution. This approach has been termed “rhetorical reasoning”, with a focus on determining the crux of any specific matter. Therefore, casuistry is not an ethical theory nor is it a deductive approach; “[r]ather, it is a case-based approach in which an argument is developed by comparing the case at hand with paradigm cases in which it is reasonably clear what course of action should be taken” [36]. It focuses on morally relevant factors in each case without claiming certain conclusions [36]. Such approaches have been described in the context of medical ethics and clinical reasoning or deliberation of choices in patient care [37–41]. Using casuistical case analysis and analogical reasoning rather than more traditional, theory-driven methodologies actually resemble common law traditions prevalent in much of the English-speaking world and, we suggest, can generate insights relevant to applied ethics and jurisprudence in the context of the current pandemic [42]. In this article, the cases that will be explored to ascertain whether it is acceptable to require compulsory vaccination against COVID-19 are the cases for (1) compulsory vaccination for common childhood diseases such as measles, mumps, rubella, and polio and (2) compulsory treatment for infectious disease.

## Compulsory vaccination for infectious diseases in childhood such as measles, mumps, rubella, and polio

Perhaps the most obvious case study to explore is that of compulsory vaccination for common diseases in childhood such as measles, mumps, rubella, and polio. Safe, effective vaccinations are widely available, with relatively good uptake in countries such as Ireland [43], England [44], and Wales [45], and those without vaccination are generally protected through herd immunity. However, vaccination uptake is dropping in Europe [46]. Consequently, there have been a number of outbreaks of these diseases in recent years throughout developed countries where effective vaccines are widely available. For example, in Ireland, there were 278 cases of mumps in the first 6 weeks of 2019, which amounted to a 545% increase on the same period in the previous year, with 60% occurring in young people, the cohort most likely affected by the since-discredited research published by Andrew Wakefield in the early 1990s linking the MMR vaccine with autism [47].

The arguments often posited in favour of compulsory vaccination in this circumstance stress the societal cost of the diseases in question, including the cost of healthcare, and the personal impact of the disease, including illness and death. This is especially so when “herd immunity” is compromised, and outbreaks occur amongst vulnerable members of society, including those who cannot receive the vaccine due to immaturity, allergies, or illness. Despite this, it is relatively uncommon for compulsory vaccination to be employed to date in western democracies.

While there are similarities between compulsory vaccination for common diseases in childhood, the analogy breaks down when one considers the scale of the impact of COVID-19, including the impact on the world economy, the unprecedented restrictions on personal liberty that have been imposed including constraints on travel within and across borders, mandatory quarantine, limits on social engagement and interaction, the death toll, and the emerging evidence of long-term health implications for a proportion of people infected with COVID-19 [48]. It is also suggested that global pandemics of this nature and their inevitability in the future represent a threat to national security “requiring a suitable infrastructure with which to tackle future outbreaks. Deployment or development of vaccination has an integral strategic role. The infrastructure and its public acceptance affect the political boundaries between national security and public health and affect the resilience of rights to refuse vaccination” [4].

This new problem, relating to infectious disease pandemics involving uncommon pathogens, cannot be considered analogous to our experience with compulsory vaccination against common infectious diseases in childhood in modern times. Indeed, the potential benefits of vaccination for children may be marginal. Further it has been suggested that the arguments in favour of adult compulsory vaccination against COVID-19 are stronger than those in support of vaccination in childhood. However, in light of expedited vaccine development, regulatory approvals and reports of adverse event incidence (e.g. myocarditis following mRNA vaccine administration [49] and unusual blood clotting despite low platelets [50]), any such programme would require comprehensive pharmacovigilance.

## Compulsory treatment of infectious disease

Compulsory treatment is another tactic utilised in public health strategies against infectious disease proliferation. The World Health Organization suggests that “[p]ublic health laws should authorise compulsory treatment only in circumstances where an individual is unable or unwilling to consent to treatment, and where their behaviour creates a significant risk of transmission of a serious disease” [51]. Any such treatment should impact upon liberty only to the extent necessary to minimise the risk to the public [51].

While compulsory treatment is utilised rarely in the context of infectious disease, it may be necessary in some circumstances to limit contagion. For example, in the USA, the Model State Emergency Health Powers Act [52, 53] provides for a variety of mandatory measures including physical examination, testing, treatment, quarantine, and isolation in the event of a bio terrorist attack or the outbreak of a natural disease [51, 54]. In South Africa, the National Health Act states that treatment without consent is not permissible unless a “failure to treat the user, or group of people which includes the user, will result in a serious risk to public health” [55].

Compulsory treatment is similar to compulsory vaccination in that both involve an invasion of bodily integrity; both should be employed in limited circumstances when the interests of public health demand it. Several of the vaccines that have been developed for COVID-19 are viral vector vaccines containing a weakened version of a live virus, while mRNA vaccines code for the spike protein of the SARS-CoV-2 virus. Therefore, similar to compulsory treatment, compulsory vaccination is a weighty step which involves compromise of bodily integrity.

However, there are important caveats. It is reasonable to argue that compulsory vaccination is generally less invasive than compulsory treatment, reflected in the rarity with which compulsory treatment is employed. The side effects of vaccinations are generally mild and serious adverse reactions rare [56]; such effects are also generally short-lived. In contrast, compulsory treatment is likely to be more invasive and may involve sustained therapeutic management over an extended period with potential for significant impact on the recipient.

Therefore, if compulsory treatment is considered acceptable in some limited circumstances, it seems reasonable to argue that compulsory vaccination against COVID-19 may also be warranted due to the reduced impact on bodily integrity and the considerable risk of COVID-19, both to population health and social and economic life, particularly if future mutations emerge which pose a greater threat to public health and social and economic life. However, public support or even acceptance will require provision of accessible and understandable information (as, e.g. explaining generic medicines and their use [57]), and may involve lengthy political ratification processes (e.g. referenda), contingent upon individual nations’ constitutions or governmental procedures.

## Conclusion

It is evident that the challenges presented by COVID-19 are novel in modern times and will influence the world economy and society for many years. Application of casuistry, or rhetorical reasoning, in “the practice of setting general laws on the basis of exceptional cases” has been criticised by the current highest ranked Jesuit, Pope Francis, albeit in a context different to the COVID-19 pandemic. Irrespective, our choice of utilising casuistical case analysis has highlighted that while more holistic solutions may be preferred, the novel nature of this pandemic requires innovative approaches to public health, which might eventually include compulsory vaccination of those who refuse vaccination. Of course any such measure would have to be limited in scope and application with procedural protections vital [58]. It is clear that there are times when the state must take firm action to prevent or mitigate risk of contagion in the interests of public health, particularly in the context of a prolonged and deadly pandemic [59]. Framed in that way, coercive measures such as compulsory vaccination might become acceptable, even if only temporarily and perhaps through exhaustion of populations and economies affected by serial outbreaks over an extended period. Indeed, it seems prudent that understandable education programmes be implemented to inform the public of public health measures, their necessity, and the potential consequences of their implementation or absence. If effective, such programmes may elicit support across all sectors of society rendering coercion unnecessary. Such support, however, is aspirational, and perhaps the most likely scenario is passive acceptance rather than active opposition based on understanding of infection risks and, hence, reduced antipathy towards vaccination.

## Declarations

### Data sharing

No additional data are available.

### Public and patient involvement

Patients were not involved in this manuscript.
